# Collagen peptides affect collagen synthesis and the expression of collagen, elastin, and versican genes in cultured human dermal fibroblasts

**DOI:** 10.3389/fmed.2024.1397517

**Published:** 2024-05-01

**Authors:** Stephan Dierckx, Milagros Patrizi, Marián Merino, Sonia González, José L. Mullor, Reyhan Nergiz-Unal

**Affiliations:** ^1^Tessenderlo Innovation Center, Tessenderlo Group NV, Troonstraat, Brussels, Belgium; ^2^PB Leiner, Part of Tessenderlo Group, Troonstraat, Brussels, Belgium; ^3^Bionos Biotech SL., Biopolo La Fe - Hospital La Fe (Torre A) Av. Fernando Abril Martorell, Valencia, Spain

**Keywords:** collagen hydrolysate, fibroblast, skin aging, antiaging, collagen

## Abstract

**Background:**

Collagen is one of the major proteins of the skin and it is particularly important for its strength and resilience. Skin aging is a natural process that is characterized by the decrease and fragmentation of collagen in the dermis. Oral supplementation with collagen peptides has been clinically shown to have a positive effect on the skin condition. However, the mechanisms of aging-related changes synthesized by cells exposed to collagen are currently not well understood. Therefore, in this *in vitro* study, the mechanisms associated with collagen, elastin, and versican in human dermal fibroblasts were investigated after exposure to collagen peptides.

**Methods:**

The effects of different concentrations of collagen peptides on cell viability and metabolism were analyzed. For gene expression analysis, human dermal fibroblasts were treated with collagen peptides. This was then followed by RNA extraction and DNA synthesis. Gene expressions of collagen type 1 (COL1A1), elastin (ELN), and versican (VCAN) were quantified by quantitative reverse transcription polymerase chain reaction (RT-qPCR). In addition, collagen levels were analyzed by confocal scanning laser microscopy using immunostaining.

**Results:**

Collagen peptides tested in the study increased the expression of the relevant COL1A1, ELN, and VCAN genes in human dermal fibroblasts (*p* < 0.005). Furthermore, confocal microscopy showed increased collagen expression in the dermal fibroblast culture after treatment with the collagen peptides (*p* < 0.005).

**Conclusion:**

These data provide cell-based evidence for the beneficial effects of exposure to collagen peptides on the skin’s collagen content and on the molecules that provide firmness and elasticity. This may support the hypothesis that collagen peptides are important for maintaining extracellular matrix (ECM) structure and skin regeneration.

## Highlights

- Fibroblasts and extracellular matrix in the skin are modulated by exposure to collagen peptides.
- Collagen peptides increase the skin’s collagen content, firmness and elasticity-related molecules.
- The potential beneficial effects of collagen peptides on skin firming and elasticity are determined.
- Exposure to collagen peptides may maintain structure of the dermis and extracellular matrix.

## Introduction

Skin aging is a natural phenomenon that is triggered by both intrinsic (genetic and chronological) changes and extrinsic (environmental and lifestyle) damage (1). A significant portion of these changes occur in the dermis, which consists largely of fibrillar collagen that forms the extracellular matrix (ECM). Dermal ECM provides mechanical stability and forms a scaffold that supports the functions of dermal cells (2, 3). Fibroblasts attach directly to collagen fibrils, and this attachment allows fibroblasts to achieve a firm morphology through opposing mechanical forces of the cytoskeleton (2, 4, 5). Structural matrix proteins such as collagen, elastin, and versican, which are produced by fibroblasts, are essential components for skin support and elasticity (3–5). Within the dermal ECM, aging is associated with a progressive loss and fragmentation of dermal collagen fibrils. This leads to thinner and structurally weakened skin (6, 7). This could be predominantly due to the decreased synthesis and increased degradation of collagen I (which is the main type of collagen) in the dermis, which is encoded by the COL1A1 gene, and increased fibril fragmentation (6), or increased degradation of collagen and elastin fibers, which is encoded by the ELN gene (7). However, many other ECM proteins besides collagen and elastin are involved in the skin structure and premature aging, such as versican, lumican, fibronectin 1, fibrin, laminins, integrins, etc. Versican is a chondroitin sulfate proteoglycan of the ECM found in a wide variety of human tissues and it is encoded by the VCAN gene. Its highly interactive nature is the basis for the important role it plays as a structural molecule that creates loose and hydrated matrices by interacting either directly with cells or indirectly with molecules that associate with cells, in part to regulate cell adhesion and survival, cell proliferation, cell migration, and ECM assembly (8, 9).

Limited *in vitro* studies have demonstrated the potential benefits of collagen peptides in regard to increasing fibroblast proliferation (10, 11). In response, cells such as fibroblasts may produce collagen although regulation of this mechanism is not yet fully understood. In light of the science behind the skin health benefits offered by collagen peptides, fibroblasts are the most common type of cell found in connective tissue and they are used to maintain the structural framework of the skin. Furthermore, they also play an important role in skin regeneration (12). Therefore, the aim of this study was to distinguish the skin antiaging potential of collagen peptides by evaluating their effect on COL1A1, ELN and VCAN gene expressions and collagen synthesis in dermal fibroblasts.

## Materials and methods

### Cell culture

Normal human dermal fibroblast cells were purchased (NHDFn, Gibco, Ireland) for use in cell viability and gene expression assays. They were obtained from neonatal (14 days or less) foreskin. Cells were grown in DMEM high glucose that contained 10% fetal bovine serum (FBS), 1% penicillin–streptomycin and 2% L-glutamine (growth medium), and in a humidified atmosphere with 5% CO_2_ at 37°C. The cells were sub-cultured every 7 days and were used in passages 5–7.

### Cell viability and proliferation

The standard methyl thiazolyl tetrazolium (MTT) assay was used to measure cell viability, proliferation, and cytotoxicity. This colorimetric assay is based on the reduction of a yellow tetrazolium salt (3-(4,5-dimethyl thiazol-2-yl)-2,5-diphenyl tetrazolium bromide or MTT) to purple formazan crystals by metabolically active cells. The viable cells contain NAD(P)H-dependent oxidoreductase enzymes that reduce the MTT to formazan. The insoluble formazan crystals are dissolved using a solubilizing solution and the resulting-colored solution is quantified by measuring the absorbance by a microplate reader. The darker the solution, the greater is the number of viable, metabolically active cells (13, 14).

For the MTT assay, fibroblast cells were cultured overnight at a density of 10,000 cells/well density in a 96-well plate. After 24 h, the cells were treated with different concentrations of SOLUGEL® collagen peptides (PB Leiner, part of Tessenderlo Group NV, Belgium) (3, 1, 0.3, 0.1, 0.03, 0.01, 0.003, and 0.001% w/v diluted in a growth medium). After 24 h of incubation, the medium was removed and the wells were washed with phosphate-buffered saline (PBS), and a 1:11 solution of MTT was added to each well. Plates were incubated at 37°C for 3 h. The MTT reagent was carefully removed, and dimethyl sulfoxide (DMSO) 100% was added to each well to solubilize formazan crystals prior to absorbance measurements at 550 nm and a reference of 620 nm. The MTT assay was set with 8 technical replicates per condition and 16 for control.

### Collagen peptides

The collagen peptides evaluated in this study (SOLUGEL®, PB Leiner, part of Tessenderlo Group NV, Belgium) were tested on normal human dermal fibroblast cells in a culture. Hydrolyzed collagen is deemed to be safe and is considered Generally Recognized as Safe (GRAS) by the Food and Drug Administration (FDA) (Ref: GRAS SCOGS report # 58 [SCOGS 58 Gelatin]). In addition, the FDA has a GRAS notification for hydrolyzed animal proteins that include hydrolyzed gelatin/polypeptides (Ref: 21 CFR 184.1553 Peptones). The GRAS status of hydrolyzed collagen is affirmed via these references. SOLUGEL® collagen peptides is produced from collagenous tissues by hot water extraction. After certain purification steps, the collagen goes through a highly controlled standardized enzymatic hydrolysis process to end up with a molecular weight between 0.3 and 8 kDa and with an average molecular weight of 2-3 kDa. Collagen peptides are characterized by a high content of glycine (23–24%), hydroxyproline (12–13%), proline (13–14%), glutamic Acid (9–10%), arginine (7–8%), and alanine (8–9%).

### Gene expression quantification

RNA isolation and quantitative reverse transcriptase polymerase chain reaction (RT-qPCR) were performed to determine gene expressions of COL1A1, ELN, and VCAN. Primer sequences are shown in Table 1. α-actin (ACT) was used as housekeeping gene. The RT-qPCR can readily produce more than one million copies of a specific DNA or RNA sequence in a simple three-step cycling process. In brief, the initial step involves the denaturation of double-stranded DNA to separate the complementary strands. From there, the second step allows for the annealing of primers to the dissociated DNA strands. Thirdly, the primers participate in an extension reaction that is catalyzed by a thermostable DNA polymerase, and the cycle is then repeated (15).

**Table 1:** The nucleotide primer sequences used in RT-qPCR.

Gene	Sequence
α-actin (ACT)	5’-CCATGCCCACCATCACGC 5’-CACAGAGCCTCGCCTTTG
Collagen 1 (COL1A1)	5’-TGGGCTGAGTGGGGTACA 5’-TGACCCCAACCAAGGCTG
Elastin (ELN)	5’-GGTGGCTTAGGAGTGTCTGC 5’-CACCTACACCTGGAGCCTTG
Versican (VCAN)	5’-CTGGTCTCCGCTGTATCCTG 5’-ATCGCTGCAAAATGAACCCG

Fibroblast cells were cultured in a growth medium at a 200,000 cells/well density in a 6-well plate, in growth media. After 24 h, the culture medium was replaced with a new culture medium supplemented with SOLUGEL® collagen peptides at 0.01 and 1%, and the untreated control samples were maintained in a culture medium. After 24 h of incubation, the medium was removed and the cells were washed with PBS. Total RNA was extracted using the miRNeasy kit (Qiagen) and its quality and quantity were checked using a Nano-Drop spectrophotometer and 500 ng of total RNA was used for cDNA synthesis. The suitability of each primer pair used in this study for RT-qPCR, ACT, COL1A1, ELN, and VCAN was previously evaluated to determine melting curves, amplification efficiency, and specificity of the primers. Finally, quantitative PCR was performed on a real-time PCR machine (Applied BioSystem ViiA7). The Pfaffl method was used to calculate the gene relative expression ratio of the gene to ACT (internal control-housekeeping gene) (16). The qPCR assay was set with four technical replicates.

### Analysis of collagen levels by confocal microscopy

Collagen levels were analyzed by confocal scanning laser microscopy using immunostaining on a monolayer cells (17). In this method, fibroblast cells were first cultured on a coverslip during a 24-h period of incubation and the medium was then replaced with a fresh medium containing SOLUGEL® collagen peptides at 0.01 and 1% w/v. Untreated control samples were maintained in the culture medium. After the incubation period, cells were washed and fixed in 4% paraformaldehyde (PFA) for 15 min. After fixation, the cells were treated with hot citrate buffer (100°C) for antigen exposure for 20 min and blocked with 10% fetal bovine serum (FBS) in PBS + 0.1% Triton X-100 for 1 h. The blocking solution was changed by anti-rabbit pAb to collagen I (ab34710, Abcam) at 1:350 in a blocking solution, overnight at 4°C. The antibody was washed and samples were stained with Alexa 488 anti-rabbit (A-11008, Invitrogen) at 1:500 in blocking solution for 1 h in the dark. At the end of the incubation period, cells were finally stained with 4′,6-diamidino-2-phenylindole dihydrochloride (DAPI) and mounted on the slide with a fluorescence mounting medium. The samples were then imaged for blue (DAPI: cell nucleus) and green (collagen) fluorescence on a Leica confocal microscope SP5 with a 40× objective (Leica, Germany). For all images, the laser excitation intensity and signal amplification values were maintained and normalized to the untreated control (17). Collagen fluorescence mean value for each image was calculated using the Leica Imaging software LAS X and 5 technical replicates per condition were used.

### Statistical analysis

Data are presented as mean values per time point, with standard error in graphs. Statistical analysis was performed by the Bionos laboratory using GraphPad Prism software (GraphPad, San Diego, CA, USA). Homogeneity between groups at baseline was tested by ANOVA followed by a Tukey/Dunnett test or Student’s t-test. Within-group differences over time and between-group differences were calculated by ANOVA with a post-hoc analysis using the Tukey or Dunnett tests or two-tailed Student’s *t*-test. Statistical significance was considered when *p* < 0.05.

## Results

### Cell viability

**Figure 1:**
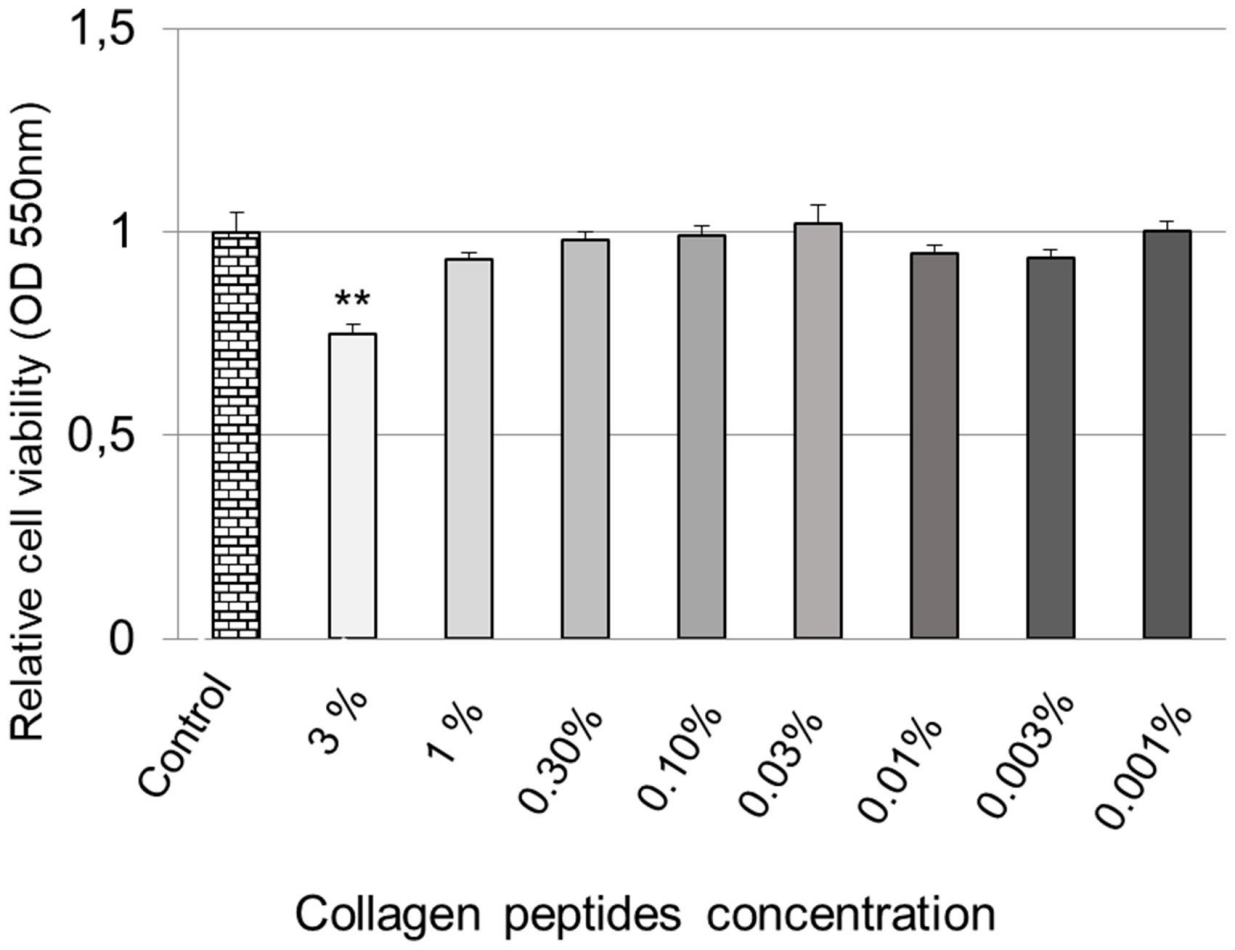
Cell viability in human fibroblasts treated with collagen peptides. A graphical representation of cell viability after treatment with different concentrations of collagen peptides for 24 h compared to the untreated control. All data are expressed as mean ± standard error ** Represents statistical significance with *p* value <0.0001.

A cell viability assay was performed on human normal dermal fibroblasts to determine the overall health and survival of the cells after treatment with collagen peptides for 24 h. Following this assay, the two non-cytotoxic concentrations were determined for use in subsequent experiments. Cell viability experiments indicated that treatment with collagen peptides did not show a relevant decrease in cell viability when applied at concentrations equal to or less than 1%; as shown in Figure 1.

### Gene expressions

**Figure 2:**
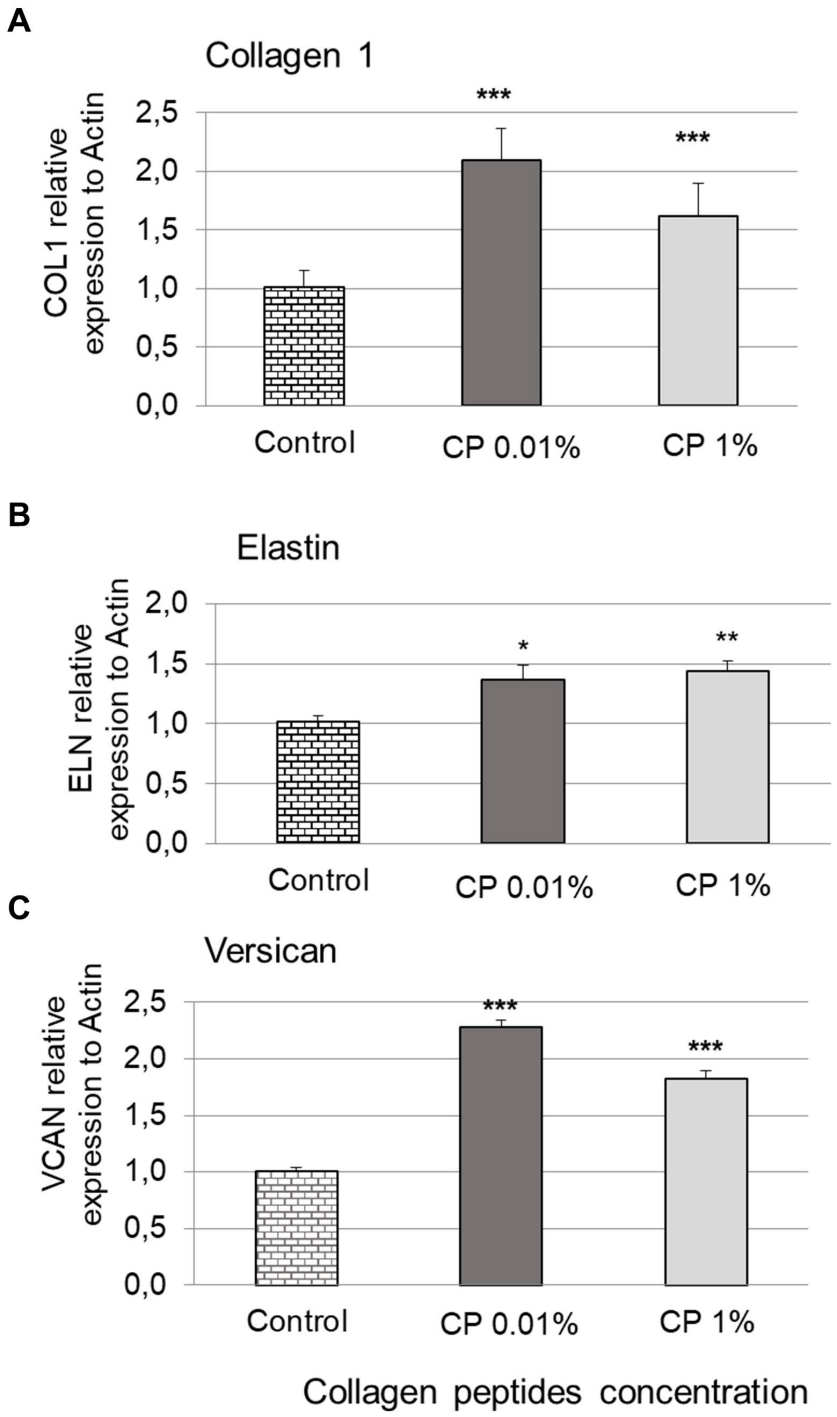
Gene expression levels. Bar graphs showing the expression results of COL1A1 **(A)**, ELN **(B)**, and VCAN **(C)** after treating normal human dermal fibroblasts (NHDF) for 24 h with collagen peptides (CP) at 0.01 and 1%, compared to the untreated control. Represents statistical significance with *p value <0.05. ** *p* value <0.01. *** *p* value <0.0001.

According to the MTT results, the 0.01% (100 μg/mL) and 1% (10,000 μg/mL) concentrations were selected for further gene expression analysis (Figure 2). Gene expression levels of COL1A1 showed that treatment of human normal dermal fibroblasts with collagen peptides at 0.01% or 1% for 24 h significantly increased *COL1A1* gene expression by 108.4 ± 7.6% and 60.5 ± 7.9%, respectively, compared to the untreated control; as shown in Figure 2A.

For ELN expression levels, treatment with collagen peptides at 0.01% or 1% significantly increased *ELN* gene expression by 35.2 ± 13.2% and 42.1 ± 10.1%, respectively (Figure 2B). Expression levels of VCAN showed that treatment with collagen peptides at 0.01% or 1% significantly increased *VCAN* gene expression by 127.6 ± 7.0% and 81.2 ± 8.4%, respectively, compared to the untreated control; as shown in Figure 2C.

### Collagen levels by confocal microscopy

**Figure 3:**
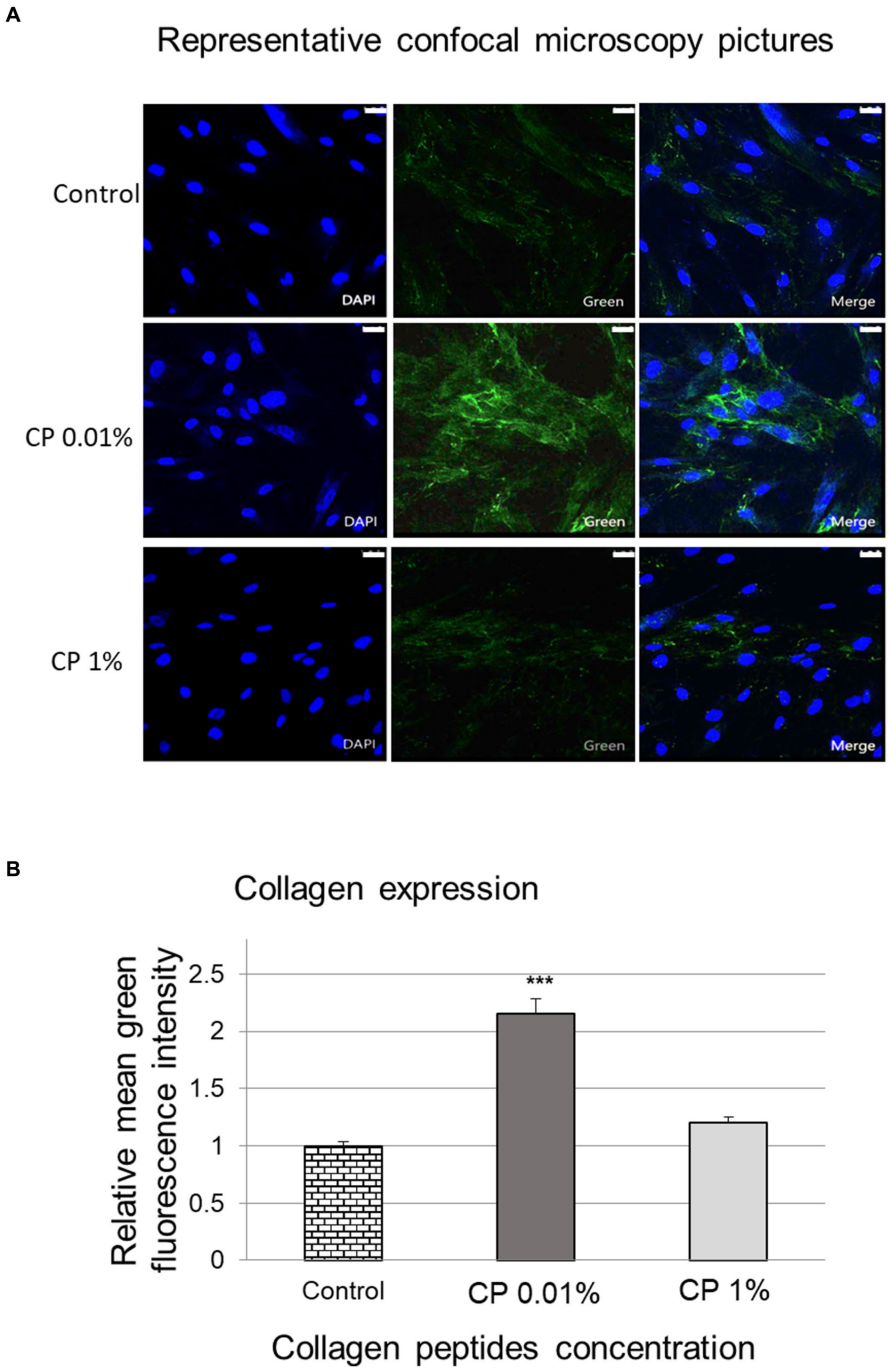
Imaging of collagen by confocal microscopy. Representative confocal microscopy images (scale bar = 25 μm) showing dermal fibroblasts (blue) and collagen fibers (green) **(A)**. The bar graph shows the mean fluorescence intensity of collagen fibers obtained from the confocal microscopy **(B)**, after treatment of normal human dermal fibroblasts with collagen peptides at 0.01 and 1% for 24 h compared to the untreated control. *** Represents statistical significance with *p* value <0.0001.

In normal human dermal fibroblasts (blue), collagen fibers (green) can normally be detected surrounding the cell nuclei as NHDF endogenously express procollagen. The results showed that treatment with collagen peptides at 0.01 and 1% for 24 h significantly increased collagen synthesis compared to the untreated control (Figure 3A).

When the mean intensity of collagen fluorescence was analyzed for each of the images in each of the conditions tested, the results showed that treatment for 24 h with collagen peptides at 0.01% concentration significantly increased collagen levels by 115.4 ± 13.2%, whereas treatment at 1% increased collagen levels by 20.2 ± 6.0%, although the results were not statistically significant (*p* > 0.05), compared to the untreated control (Figure 3B).

## Discussion

Collagen is the main structural protein in the extracellular space of the various connective tissues in the human body. As the main component of connective tissue, it is the most abundant protein in humans and it accounts for 25–35% of total body protein content (5). As fibroblasts are the most abundant collagen-forming cells, this study was based on fibroblasts and the possible skin aging-related molecules they express (4). Therefore, this study has the potential to demonstrate that collagen peptides can promote their skin anti-aging capacity by altering dermal fibroblast collagen synthesis *in vitro* and enhancing collagen, elastin, and versican gene expressions.

In this study, the cell viability results indicated that treatment with collagen peptides at a concentration equal to or less than 1% did not show a relevant decrease in cell viability. No positive dose-dependent effect was observed in the concentration range studied. In other words, higher collagen peptide concentrations in the culture media did not result in higher relative gene expression (12). This observation might indicate that collagen peptides are more effective when used at lower concentrations.

Gene expression of COL1A1 contributes to the collagen synthesis (18). Within the dermal ECM, aging is associated with a thickening of collagen fibrils and the disorganization of the total collagen content (6, 7). This is mainly due to decreased procollagen I synthesis, which is encoded by the COL1A1 gene, and increased fibril fragmentation (6) or increased degradation of collagen and elastin fibers, which is encoded by the ELN gene (7). However, many other ECM proteins besides collagen and elastin are involved in the skin structure and premature aging, such as versican (8). The collagen peptides tested in the study increased the expression of the relevant COL1A1, ELN, and VCAN genes in human dermal fibroblasts (*p* < 0.005). Some studies have reported increased collagen synthesis and COL1A1 expression in fibroblasts after treatment with collagen peptides (11, 18). The results on COL1A1 expression levels provide additional evidence to support the use of collagen peptides as a food supplement with beneficial effects on the skin, mainly due to the fact that it compensates for the loss of collagen, which is one of the causes of skin aging (1, 19, 20).

Elastin is an important protein of the ECM and it is largely responsible for skin elasticity and the mechanical strength of collagenous tissues (6, 21). As there is limited evidence to show the effect of collagen peptides on altering elastin expression levels, these results are insightful in terms of understanding the benefits of collagen peptide supplementation in the skin (6). In contrast, in one study, collagen-derived hydroxyproline containing peptides did not alter the expression of matrix-related COL1A1 and ELN genes in cultured dermal fibroblasts (20). In several clinical studies, it has been demonstrated that the oral ingestion of hydrolyzed collagen increased the elasticity of the skin (22–24) and this positive effect might be partly due to the increase in *ELN* expression in dermal fibroblasts that is observed in this *in vitro* study.

Versican is a large chondroitin sulfate proteoglycan that is important due to its ability to form loose and hydrated matrices (8). Versican expression levels showed a significant increase following treatment with collagen peptides compared to the untreated control (*p* < 0.005). Correspondingly, according to a few of *in vitro* studies undertaken, collagen peptides may stimulate cell proliferation and glycosaminoglycan production in cultured human dermal fibroblasts (20, 25, 26) and may enhance the formation of stable dermal fibroblast-derived extracellular matrixes (10). Significantly, this data is unique in terms of demonstrating the direct relationship between collagen peptides and versican expression.

The hypothesis that collagen peptide supplementation in clinical trials has the potential to stimulate fibroblast activity raises the question of whether it may also be directly responsible for increased collagen synthesis (27–29). Skin condition is largely dependent on the homeostasis of the dermal extracellular matrix, which is primarily defined by collagen and related fibers and their network (11, 18). The dermis contains large amounts of ECM components such as collagen and glycosaminoglycans that are mainly produced by fibroblasts (30). In this *in vitro* study, higher collagen expression was detected by confocal microscopy in the dermal fibroblast culture after treatment with the collagen peptides (*p* < 0.05). In one study, collagen peptide supplementation induced increased fibroblast density and enhanced collagen fibril formation in the dermis (26). There are a few examples in literature where fibroblasts have been induced by different types of collagen peptides (18, 31). Nevertheless, there is limited data showing confocal imaging for increased collagen synthesis by fibroblasts.

A complex network of interlaced collagen fibrils in the dermis provides support to the epidermis, and together with elastin and microfibrils, it gives the skin its elasticity and resilience through fibroblast proliferation and matrix protein synthesis (10, 25). Not only triggering the collagen synthesis but also the inhibition of degradation proteins (32) or changing matrix protein synthesis (10) may be responsible for this effect. In a study involving collagen content being measured in fibroblasts, the increase in collagen content was attributed to stimulation of biosynthesis and decreased collagen I metabolism through the inhibition of metalloproteinase activity (MMP) 1 and 2 (32). As the fibroblasts are the most abundant collagen-producing cells, these results showing an increase in collagen synthesis in dermal fibroblasts reinforce the scientific evidence that supports the effect of collagen peptides on skin aging due to irreplaceable collagen loss (4, 5, 12). Therefore, the *in vitro* treatment with collagen peptides for 24 h on NHDF displays on the skin’s collagen content and on the molecules that provide firmness and elasticity. This trend should be further investigated to understand the basis of this behavior, which might offer a key finding from a clinical perspective. Several scientific and clinical studies have shown the importance of supplementing a healthy and balanced diet with hydrolyzed collagen to improve skin properties (27, 28). This supplementation would stimulate the growth of fibroblasts, which are responsible for collagen synthesis, as well as the synthesis of elastin and glycosaminoglycans, which would make it possible to improve the hydration, elasticity, and appearance of the skin.

## Conclusion

This is a preclinical study suggesting the beneficial effects of pure SOLUGEL® collagen peptides on skin conditions. The novel results of this study suggest that fibroblasts and ECM in the skin are modulated by collagen peptides. The underlying mechanism and the effects of collagen peptides on cell signaling and modulation of ECM proteins using *in vitro* cultured epidermal fibroblasts and keratinocytes require further investigation. All in all, these data provide scientific, cell-based evidence for the potential beneficial effects of the exposure of collagen peptides on skin-firming and anti-aging properties, which suggests that it may help to maintain the structure of the dermis and ECM.

## Data availability statement

The raw data supporting the conclusions of this article will be made available by the authors, without undue reservation.

## Author contributions

SD: Conceptualization, Funding acquisition, Investigation, Methodology, Project administration, Resources, Supervision, Validation, Writing – review & editing. MP: Conceptualization, Funding acquisition, Investigation, Methodology, Project administration, Resources, Supervision, Validation, Writing – review & editing. MM: Conceptualization, Data curation, Formal analysis, Investigation, Methodology, Writing – review & editing. SG: Formal analysis, Investigation, Methodology, Writing – review & editing. JM: Conceptualization, Data curation, Formal analysis, Methodology, Resources, Supervision, Validation, Writing – review & editing. RN-U: Conceptualization, Funding acquisition, Investigation, Visualization, Writing – original draft, Writing – review & editing.
